# A Cost of Sexual Attractiveness to High-Fitness Females

**DOI:** 10.1371/journal.pbio.1000254

**Published:** 2009-12-08

**Authors:** Tristan A. F. Long, Alison Pischedda, Andrew D. Stewart, William R. Rice

**Affiliations:** Department of Ecology, Evolution, and Marine Biology, University of California Santa Barbara, Santa Barbara, California, United States of America; University of New South Wales, Australia

## Abstract

Adaptive mate choice by females is an important component of sexual selection in many species. The evolutionary consequences of male mate preferences, however, have received relatively little study, especially in the context of sexual conflict, where males often harm their mates. Here, we describe a new and counterintuitive cost of sexual selection in species with both male mate preference and sexual conflict via antagonistic male persistence: male mate choice for high-fecundity females leads to a diminished rate of adaptive evolution by reducing the advantage to females of expressing beneficial genetic variation. We then use a *Drosophila melanogaster* model system to experimentally test the key prediction of this theoretical cost: that antagonistic male persistence is directed toward, and harms, intrinsically higher-fitness females more than it does intrinsically lower-fitness females. This asymmetry in male persistence causes the tails of the population's fitness distribution to regress towards the mean, thereby reducing the efficacy of natural selection. We conclude that adaptive male mate choice can lead to an important, yet unappreciated, cost of sex and sexual selection.

## Abstract 2

Females are frequently harassed and harmed by males attempting to obtain matings. When these males are also “choosy” with their courtship, there may be negative consequences to the species' ability to adaptively evolve.

## Author Summary

In many species, females are frequently subject to harassing courtship from males attempting to mate with them. These persistent male behaviors can result in females incurring substantial direct fitness costs. We set out to examine how these costs may influence adaptive potential in a species that also exhibits male mate choice, i.e., a preference by males for females exhibiting certain traits. We found that harmful courtship behaviors were directed predominantly towards females of greater reproductive potential (and away from females of lesser potential), resulting in a reduction in the variation of lifetime reproductive successes among females in the population. This change in distribution of realized fitnesses represents a previously unappreciated consequence of sexual conflict–adaptive male mate preference can slow the rate of accumulation of beneficial mutations and speed the rate of accumulation of harmful mutations, thereby creating a “sexual conflict adaptive load” within a species.

## Introduction

Historically, most studies of mate choice have focused on mate preference by females, because this sex typically has higher levels of parental investment and lower variance in realized fitness [1]–[4]. Mate choice by males, however, is a common feature of many species [5]–[8], yet its adaptive consequences are far less commonly considered [7],[9], and are typically only studied in species with reversed sex roles [10]–[12]. Here, we focus on species with “typical” sex roles that also experience sexual conflict due to antagonistic male persistence (e.g., unrelenting courtship and repeated mating attempts) that arises because the optimal outcomes of mating interactions often differ for males and females [13],[14]. It is well established that females can suffer substantial fitness costs from receiving too much male attention [15]. For instance, male sexual persistence is an important source of female mortality and/or reduced fecundity in a number of species, e.g., frogs, *Crinea georgiana* [16]; toads, *Bufo bufo* [17]; feral sheep, *Ovis aries* [18]; lizards, *Lacerta vivipara* [19]; ducks, *Anas platyrhynchos* [20]; orangutans, *Pongo pygmaeus* [21]; water striders, *Gerris odontogaster* [22]; and fruit flies, *Drosophila melanogaster* [23],[24]. In this study, we focus on a different harmful consequence of females being subject to male persistence that only occurs when males evolve a mate preference for high-fecundity females: a reduced rate of adaptation.

### Model

**Figure 1:**
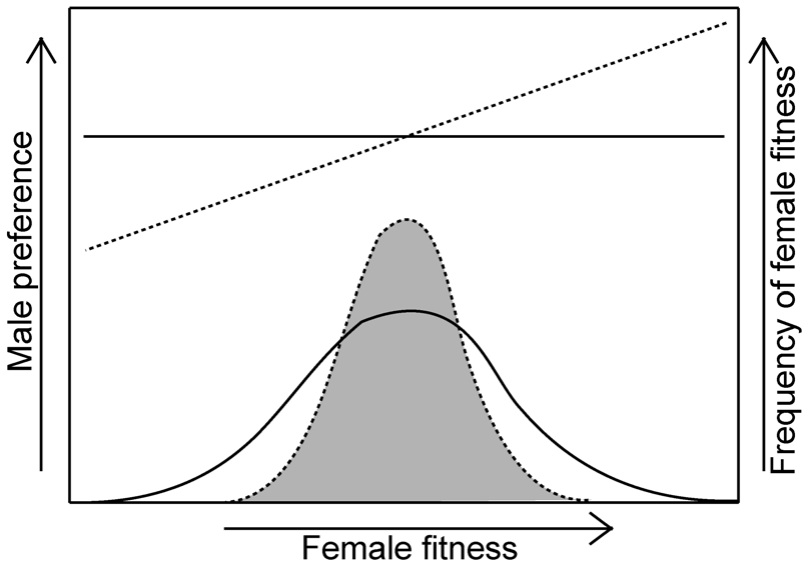
Effect of mate choice on the distribution of female fitness in species with antagonistic persistence. When males direct their antagonistic persistence more towards higher-fitness females, the tails of the female fitness distribution are predicted to regress towards the mean (dotted line and shaded distribution) compared to the case where male persistence is randomly applied to all females in the population (solid line and open distribution).

We first develop a graphical model in which a single quantitative trait is a reliable, direct indicator (rather than an indirect indicator, like a costly ornament) of a female's “intrinsic” fecundity (i.e., fecundity in the absence of costly male persistence). For example, in a wide diversity of taxa, variation in female fecundity is strongly correlated with body size [2],[4] because larger females have more resources to invest in fecundity. Henceforth, we arbitrarily assume that a female's body size is the phenotypic trait correlated with fecundity, but our logic applies to other indicator traits that directly influence her fecundity, such as parasite load [25] or abdomen size in many insects [7]. Males are expected to evolve a mating preference for larger females whenever this preference increases their own lifetime reproductive success [14]. Such an adaptive male mate preference will cause larger, intrinsically high-fecundity females, to receive more antagonistic male persistence, compared to smaller, intrinsically low-fecundity females. The fitness consequences of this relationship will depend upon how female resistance to male-induced harm scales with the indicator trait (in this case, body size). Assuming that a female's resistance to the harmful male persistence does not rise sufficiently fast with increasing body size, the male preference should reduce the fecundity of large females and increase that of small females, thereby reducing the standing variance in fitness (Figure 1). As a result, the selective advantage of any beneficial genetic variation that makes females more competitive for limiting resources, and hence more fecund, will experience a smaller selective advantage than if harmful male persistence was randomly applied to females throughout the population. Such nonrandom male persistence will cause adaptive evolution in females to be slowed whenever female fecundity is: (*i*) heritable, (*ii*) genetically correlated with the indicator trait, and (*iii*) a major determinant of her lifetime fitness that does not strongly trade off with her other fitness components. A reduced rate of adaptive evolution by females can also be deduced from Fisher's fundamental theorem [26], so long as the male-induced reduction in the phenotypic variation in female fecundity also leads to a reduction in the additive genetic variation among females. Furthermore, when there is a positive genetic correlation for fitness between females and males, adaptive male mate choice is expected to reduce the rate of adaptation in both sexes. Although a counteracting effect could occur if male preference for high-fecundity females increases the variance in male fitness, or if male preferences lead to positive assortative mating for fitness, here we focus on female fitness and the potential for male mate preferences to reduce its heritable variation.

The conclusion that adaptive male choice leads to a reduced rate of adaptation by females can also be deduced by focusing on mutations at a single arbitrary locus. Let the mutation rate to new beneficial mutations be *U*_Ben_ and the selective advantage of the mutation, expressed as a selection coefficient and averaged across the sexes, be *s*. Assuming approximate additivity (i.e., little dominance), the probability of the mutation becoming fixed can be calculated using the diffusion approximation [27] as 2*s*(*N_e_*/*N*), where *N* is the population size and *N_e_* is the effective population size. With recurrent mutation to new beneficial mutations, the rate of advance of adaptive evolution is approximated by:
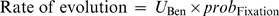
(1)

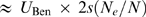
(2)


If we partition selection between the sexes and let the selective advantage of a mutation be *s_♂_* in males and *s_♀_* in females then,
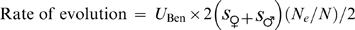
(3)

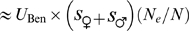
(4)


Next, we assume that the expression of the beneficial mutation also increases the attractiveness of females to males (e.g., as a result of increasing her body size), so that those females expressing the beneficial mutation receive an excess of antagonistic male persistence. We express this cost with an additional selection coefficient *s_♀_*_biased-persist_, which is applied only to females,
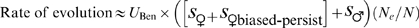
(5)


Comparison of Equations 4 and 5 demonstrates that the rate of adaptive evolution will always be slower whenever males bias their antagonistic persistence towards fitter females and cause *s* **_♀_**_biased-persist_ to be negative; i.e., when there is adaptive male mate choice and increased male persistence is harmful to females. Increased male persistence directed towards more fecund females that express the beneficial allele reduces the selective advantage of those females and thereby reduces the variance in fitness among females in the population.

### Predictions and Assumptions

The primary prediction from our models is that, in species with antagonistic male persistence, adaptive male mate preference leads to a “cost of being an attractive female.” This cost reduces the selective advantage of females expressing more beneficial genetic variation (and hence are larger, on average) and increases the fitness of females expressing less of this variation (and hence are smaller, on average). Put more simply, adaptive male mate preference causes the tails of the population's distribution of female lifetime fecundity to regress towards the mean (Figure 1). This prediction is contingent on four assumptions that must be met in order for our model to operate: (*i*) lifetime fecundity and net fitness are strongly genetically correlated, (*ii*) body size and fecundity are positively correlated, both phenotypically and genetically, (*iii*) more antagonistic male persistence is directed towards females with higher intrinsic fecundity (i.e., potential fecundity in the absence of costly male persistence), and (*iv*) female resistance to male-induced harm does not rise sufficiently fast with increasing body size. We tested the major prediction of the model, and its underlying assumptions, using a laboratory population of the model species *D. melanogaster.* In this population, assumption (*i*) is well established [28],[29], so here we focus on testing whether our population meets assumptions *ii*–*iv*, before experimentally assessing whether male mate preference for high-fitness females causes the tails of the distribution of lifetime fecundity to regress towards the mean.

## Results

### Assumption (*ii*)—A Positive Genetic and Phenotypic Correlation between Body Size and Lifetime Fecundity

**Figure 2:**
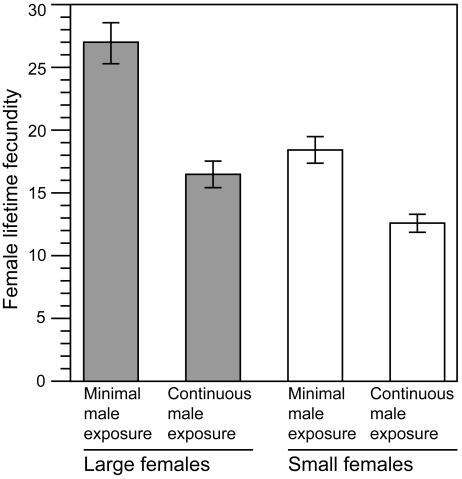
Effect of the extent of male exposure on female fecundity. Mean (±SE) lifetime fecundity (number of eggs produced) when individual large (shaded bars) and small (open bars) females experience minimal or continuous exposure to males.

Joint measures of female body size and lifetime fecundity in our base population (LH_M_) of *D. melanogaster* indicated that these two phenotypic traits are strongly correlated. As predicted from past studies of many taxa [2],[4],[30], fecundity was higher in large females compared to small females (Figure 2). This result was found both when females experienced minimal exposure to males (mean ± standard error [SE]: large females, 27.3±1.59; small females, 18.42±1.07; *t*-test *t* = 4.64, *df* = 98, *p*<0.0001; *p*-values reported throughout the manuscript are two-tailed) and when male exposure was continuous (large females, 16.5±1.06; small females, 12.62±0.72; *t*-test *t* = 3.01, *df* = 98, *p*<0.003). We tested for a genetic correlation between body size and fecundity in a separate study in which two populations each were artificially selected for either large or small body size. After 83 generations of divergent selection, lifetime fecundity was significantly higher in the lines selected for large body size compared to the lines selected for small body size (mean ± SE: large females, 31.38±1.81; small females, 15.25±1.58; *t*-test *t* = 6.69, *df* = 2, *p* = 0.02). Since body size was the only target of artificial selection, the large divergence in fecundity between treatments demonstrates a strong positive genetic correlation between body size and fecundity, a result consistent with other research [31].

### Assumption (*iii*)—Antagonistic Male Persistence Is Directed More towards Females with Higher Intrinsic Fecundity

**Figure 3:**
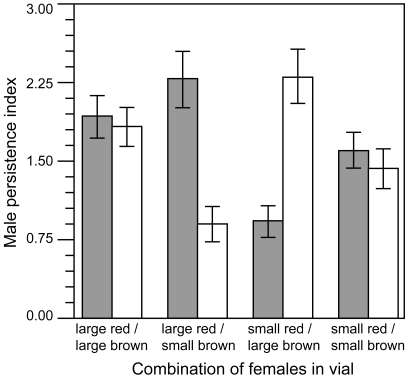
Male mate persistence towards females of similar or differing body size. Mean (±SE) number of male persistence behaviours directed towards either brown-eyed (open bars) or red-eyed (shaded bars) females of large or small body size.

**Figure 4:**
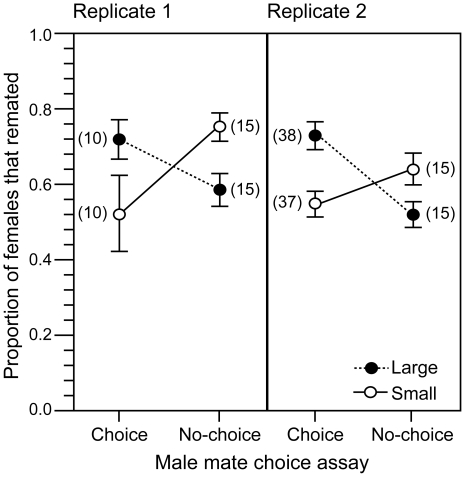
Remating rates under different male-choice environments. Mean (±SE) proportion of eight large (closed circles) or eight small (open circles) nonvirgin females that mated over a 24-h period when housed in vials with 16 males and eight similar-sized females (no-choice) or 16 males and eight randomly selected females (choice). Data are from two replicate assays. Number of replicated vials per assay is shown in brackets.

To test this assumption, we first measured how the persistence (courtship behaviour) of individual males was allocated between two nonvirgin females (differing in eye colour phenotype, brown or red, for ease of individual identification). We performed a two-way ANOVA on the amount of persistence behaviour directed towards each female, with the body size of that “target” female (large or small), the body size of the competitor female present in the test tube (large or small), the eye colour of the target female (red or brown), and all possible interactions as predictor variables. This analysis was significant overall (F_7,232_ = 7.50, *p*<0.0001), with significant effects of the target female body size (F_1,232_ = 38.37, *p*<0.0001) and the body size of the competitor female (F_1,232_ = 13.53, *p* = 0.0003), but no effects of eye colour (F_1,232_ = 0.41, *p* = 0.52), or any of the interactions (all *p*>0.60). When individual males were housed with two nonvirgin females differing in body size, males directed more persistence towards the larger female than towards the smaller female (paired *t*-tests, *p*≤0.0002, Figure 3). When males were housed with two nonvirgin females of similar body size (both small or both large), the levels of male persistence directed towards the red- and brown-eyed females were not significantly different (paired *t*-tests, *p*≥0.50, Figure 3). Further evidence of a male mate preference for nonvirgin females of larger body size was obtained from mating assays conducted under conditions that more closely mimicked the normal culture environment of the LH_M_ population (16 males combined with 16 females during the “adult competition” phase of the life cycle [28],[32]). When presented with a choice of nonvirgin females differing in body size, males mated with large-bodied females at a greater rate than with small-bodied females (generalized linear model [GLM] with binomial error terms; *p*_consensus_ = 1.17×10^−5^, Replicate 1: χ^2^_1,18_ = 5.90, *p* = 0.015; Replicate 2: χ^2^_1,74_ = 22.87, *p*<0.0001; Figure 3). These remating results are unlikely to have arisen from large females possessing a greater receptiveness to male courtship effort because males kept under “no-choice” mating conditions (where either only large or only small nonvirgin females were present) mated with small females more frequently than with large females (GLM with binomial error terms; *p*_consensus_<1×10^−6^, Replicate 1: χ^2^_1,28_ = 14.79, *p* = 0.0001; Replicate 2: χ^2^_1,28_ = 7.16, *p* = 0.0075; Figure 4).

### Assumption (*iv*)—Female Resistance to Male-Induced Harm Does Not Rise Sufficiently Fast with Increasing Body Size

To test this assumption, we compared the reduction in lifetime fecundity of large and small females when they were either minimally or continuously exposed to males (Figure 2). Continuous male exposure harmed large females more than small females (two-way ANOVA, interaction between body size and male exposure, F_1,196_ = 4.75, *p* = 0.031), indicating that larger females were not more resistant to the harmful male persistence that they received.

### Prediction—Male Mate Preference for High-Fitness Females Causes the Tails of the Distribution of Lifetime Fecundity to Regress towards the Mean

**Figure 5:**
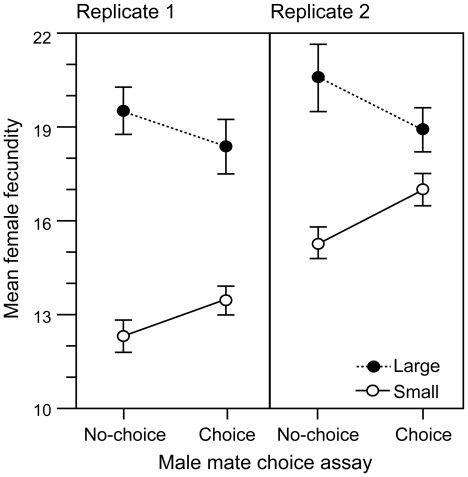
Effects of male mate persistence and choice on female fecundity. Interaction plots comparing mean (±SE) fecundities (number of eggs produced) of large (closed circles) and small (open circles) females from the two replicates of the mate choice and no-choice assays (described in legend of Figure 4).

Finally, we tested the model's key prediction by comparing the mean fecundities of large- and small-bodied females used in the choice and no-choice mating assays described earlier. In the no-choice assays, where all females were either of large or small body size, male preference for large female body size could not cause them to direct their antagonistic persistence away from smaller females and towards larger females. In contrast, in the choice assays, where females of different body sizes were simultaneously present, a redirection of antagonistic male persistence towards larger females was possible. We found that the difference in the mean fecundities of large- and small-bodied females was smaller when males could direct their antagonistic persistence towards large females (Figure 5, *p*_consensus_ = 0.012, interaction tests for each replicate: F_1,46_ = 2.79, *p* = 0.1 for the smaller, first replicate; F_1,101_ = 4.92, *p* = 0.03 for the larger, second replicate).

## Discussion

The results of our male mate preference tests clearly demonstrate that males have mate preferences for larger nonvirgin females—a result consistent with earlier work on virgin females [33]. Rather than displaying an “undiscriminating eagerness” [34] to mate, when given a choice between females differing in body size, male *D. melanogaster* preferred to court and mate with large, high-fecundity females over small, low-fecundity females. Given the significant fecundity differences associated with female body size described above, this mate preference is likely to be adaptive from the male's perspective, as mating with larger, more fecund females is likely to yield greater direct, as well as indirect [35], benefits. It is unlikely that this male mate preference is adaptive from the female's perspective, as several studies have established that chronic male persistence in the LH_M_ population is very harmful to females, and it is not sufficiently compensated by indirect genetic benefits [36]–[38]. Our experiments demonstrate that larger females receive more harmful male persistence but do not reveal the specific mechanism by which this harm accrues. Further work will be needed to resolve the degree to which this increased harm is due to harassment during courtship [24], damage associated with copulation [39], and/or the activity of products transferred in the male's seminal fluid [23],[40].

Having experimentally ascertained that the LH_M_ population of *D. melanogaster* satisfied all the assumptions necessary in which to test the key prediction our model, we were able to meaningfully assess the fitness consequences of adaptive male mate preferences. When males had the ability to bias their antagonistic persistence towards large-bodied females, we saw a decrease in the mean fecundity of these preferred females, compared to those large females that were in an experimental environment where all females were of similar size, and biases of antagonistic male persistence were not possible. In contrast, small-bodied females were, on average, able to realize relatively higher fecundities when they were housed with larger females (which, our study indicates, were attracting more harmful male persistence) than they were when they were housed in an environment in which males had no other choice of mates. Although our study found that males directed more courtship towards large females and also mated them more frequently, both of which can be harmful in and of themselves [24], the observed cost to large females might also have occurred because large females were mated, on average, to more harmful males [30],[41] than were smaller females. Irrespective of the mechanism of this cost, together these assays revealed how male mate preferences will ultimately cause the tails of the distribution of fecundity to regress towards the mean. Since adult lifetime fecundity is strongly correlated with lifetime fitness in females of the LH_M_ population [42], this male-driven sexual selection is expected to reduce the rate of adaptive evolution of any trait that is positively correlated with female body size. It is common for deleterious mutations to reduce body size in *D. melanogaster* [43], and it is reasonable to assume that many beneficial mutations will cause their carriers to be more competitive as larvae, allowing them to garner more resources during the larval competition phase of their life cycle and become larger, more fecund, adults. As a consequence, male mate preference for larger females is expected to commonly interfere with both progressive evolution and to increase the population's mutational load by interfering with purifying selection. For example, suppose that environmental change led to selection for alleles conferring higher desiccation tolerance. If more desiccation-tolerant females had a competitive advantage such that they grew to a larger size prior to reproduction (e.g., [44]), then a male preference for these females would reduce their relative fecundity and increase that of smaller, less desiccation-tolerant females. As a result, the population may be less responsive to environmental change, become an inferior competitor species, and be at a greater risk of extinction.

Collectively, our results support our model's key prediction that male mate preference for high-fitness females reduces the selective advantage of larger, more fecund females and increases that of smaller, less fecund females. This finding, obtained in a laboratory population, is likely to apply to natural populations for two reasons. First, the study was done on a large, outbred population that has been maintained in a competitive laboratory environment, at continuous large size, for over 400 generations [28],[32]. Over this period of time, the opportunity for adaptation to the laboratory environment should have been substantial, permitting the flies to be experimentally assayed under conditions to which they are highly adapted. Second, we measured natural variation in body size, rather than inducing extreme body size variation via nutritional deprivation and/or excessive larval crowding. This was accomplished using a sieve shaker device (developed by ADS and WRR), which enabled us to quickly sort thousands of adult flies based on natural variation in their body size, and obtain the largest and smallest individuals to use in our experiments. Flies from these two body size groups differed markedly in fecundity, with the larger females producing over 30% more eggs than small females under both minimal and continuous male exposure conditions. Although our assays of male mate preference support a directional preference for large-bodied females, in one assay (Figure 2), males could only choose between females of large and small body size. Thus, there is the possibility that the true male preference function favours females of intermediate size. However, in our second assay (Figure 3), males were able to choose between large or small females versus random females (average), and these data support the conclusion that male preference is monotonic for larger females.

Our model of adaptive male mate choice in the context of harmful male persistence has important limitations. First, we have implicitly assumed that the increased male persistence (directed toward larger, more intrinsically fecund females) does not cause larger females to have lower than average fecundity. Second, male condition may be more variable in nature compared to the laboratory, and condition-specific patterns of male persistence could either enhance or reduce the bias of male persistence toward larger females. Third, we have ignored complicating factors such as size-assortative mating interactions, e.g., smaller females receiving persistence predominantly from smaller or poor-condition males. Fourth, we have assumed that male mate choice is based on a female trait that directly influences her fecundity, such as body size. Theory predicts that this type of male mate preference will lead to a monotonic preference for larger females [45],[46]. However, when the preferred female trait is a costly indicator of fecundity, such as an energetically expensive ornament, then males can evolve to prefer intermediate trait values in females [45],[46], and our model would not apply. Fifth, our model may not apply to species where females obtain direct net benefits from increased mating rates, such as those with nuptial feeding [47]. Lastly, we have assumed a static male preference and female indicator trait. In many contexts, these two traits can be expected to coevolve, and this dynamic is not included in our model. Nonetheless, our empirical work suggests that the requisite conditions for the model to operate, at least transiently, can feasibly be achieved.

Our finding of harmful effects of adaptive male mate choice represents a previously unappreciated cost of sexual reproduction in species with antagonistic male persistence. Rather than simply showing that male-induced harm reduces overall female fecundity, we have shown that biases in the distribution of this harm among mates reduces the selection differential between females with intrinsically high and low fecundity. This reduced efficacy of natural selection will retard a population's rate of adaptive evolution and increase both its equilibrium mutational load and its stochastic accumulation of harmful mutations. The cost of adaptive male mate choice, however, only applies when males can reliably ascertain a female's fecundity using a trait that is heritable and correlated with heritable fitness variation. In *Drosophila*, female body size represents such a trait since it is influenced by both genotype [48],[49] and a number of environmental factors (including temperature, nutrition and larval crowding conditions [50]), and responds rapidly to directional selection. In species with little or no heritability for body size, however, an adaptive cost of male preference for high-fecundity females would not apply. Nonetheless, given the prevalence of male mate preferences [7], this new cost that we describe may be a widespread evolutionary phenomenon. For this reason, it should be considered in the broader context of the ongoing debates over the interfering or reinforcing role that sexual selection plays in the process of adaptation, and whether sexual selection increases or decreases the risk of extinction of populations and species [51].

## Materials and Methods

### Experimental Animals and Female Size-Sorting Technique

For all male–female interaction assays, we used *D. melanogaster* adults obtained from the wild-type LH_M_ population [28],[29] or from a replicate population (LH_M_-*bw^D^*) in which a dominant *brown-eyed* marker (*bw^D^*) had been introgressed through repeated backcrossing into the LH_M_ genetic background. The LH_M_ population is maintained on a 14-d culture cycle with a 12-h L∶12-h D diurnal cycle at 25°C in humidity-controlled incubators. Briefly, each generation begins with eggs placed in 56 “juvenile competition” vials (150–200 eggs per vial; each vial containing 10 ml of cornmeal/molasses medium). After 11.25 d, emerging adults are lightly anesthetized with CO_2_, mixed among vials, and transferred to “adult competition” vials (16 pairs of males and females per vial), which are seeded with 6.4 mg (dry weight) of live yeast. After 2 d of adult competition, the flies are transferred to “oviposition” vials, and then discarded after laying eggs for 18 h. The eggs laid in these oviposition vials are culled to a density of 150–200 eggs per vial and become the “juvenile competition” vials of the next generation. Because only eggs from the oviposition phase of the life cycle are used to propagate the next generation, and populations have been consistently maintained under these culture conditions for over 400 generations, the number of eggs laid during the 18-h oviposition phase represents a meaningful measure of lifetime fecundity in these populations. As such, experiments were designed to mimic these culture conditions as closely as possible. Detailed culturing protocols for these large populations (adults *n*>1,800 per generation for LH_M_ and *n*>1,300 per generation for LH_M_-*bw^D^*) can be found elsewhere [28],[29]).

We altered the quality of potential female mates by collecting females of differing adult body size, a phenotypic trait that is frequently positively correlated with fecundity [2],[30],[52]. We collected flies from the ends of the normal distribution of body sizes that are produced under typical lab culture conditions. Flies were sorted by size with the use of a sieve shaker device (Gilson Performer III, Gilson Company) which mechanically separates anesthetized flies on the basis of their ability to pass through a series of 20 electroformed sieves, in which the diameter of the holes in each sieve was 5% larger than the diameter of the holes of the sieve below (diameter of top sieve holes = 1,685 µm; diameter of bottom sieve holes = 800 µm). Flies were placed into the column (under light CO_2_ anaesthesia), and were agitated at a rate of 3,600 vibrations min^−1^ for 2 min. By using this technique, it was possible to quickly sort hundreds of flies simultaneously on the basis of their body size. For all experiments, “small” flies were defined as those that were small enough to pass through the 1,095-µm diameter sieve, whereas “large” flies were those that were too large to pass through the 1,281-µm diameter sieve.

### Assessment of the Phenotypic Correlation between Body Size and Fecundity in Females, and Quantification of the Relationship between Body Size and Female Resistance to Male-Induced Harm

To assess the phenotypic correlation between body size and fecundity, we collected adult flies from the LH_M_ population as they eclosed as virgins on day 9 of their life cycle. Flies were separated by sex, and on the following day, females were sorted by size using the sieve sorter protocol described above. One hundred female flies each of large and small body size were then placed individually (under light anaesthesia) into small test tubes that had been seeded with 0.4 mg of yeast (the amount of yeast per female experienced under normal culture conditions). Into each of these vials, three adult males were placed for a period of 2 h, during which time all virgin females were observed to have mated once. Males were then removed randomly from half of the vials to create 50 adult competition vials with minimal male exposure and 50 with continuous male exposure for each female body size category. Maintaining flies under these two conditions allows us to confirm that there is an intrinsic difference in fecundity between females of different sizes that is independent of the negative net fitness effects of continuous male presence. Matching the normal culturing protocol of the flies, vials were returned to the incubator for an additional 2 d, at which time flies were transferred to oviposition vials containing fresh medium (with a scored surface to encourage oviposition) for a period of 18 h before being discarded. The number of eggs laid in each vial was counted, and mean fecundities were compared using *t*-tests for females differing in size in each male-exposure treatment. The complete dataset was also used to test the assumption that female resistance to male-induced harm does not rise sufficiently fast with increasing body size, by examining whether or not female flies of one size were harmed more by continuous male exposure.

### Assessment of the Genetic Correlation between Body Size and Fecundity in Females

In order to verify that there was a genetic correlation between body size and fecundity, we assessed the fecundity of females obtained from populations of *D. melanogaster* that are part of an ongoing experimental evolution project (of ADS and WRR) in which females had been artificially selected for either large or small body size using a size-sorting procedure similar to that described above. These populations are otherwise cultured in a manner similar to the LH_M_ population from which they were all originally derived. At the time of the assay, the artificial selection had been operating for 83 generations in each of two replicate populations per treatment, and there had been considerable divergence in body size (mean female diameter [µm] ± SE: large treatment, 1,218.5±37.67; small treatment, 786.7±43.5; *t*-test *t* = 7.51, *df* = 2, *p*<0.01). For this assay, 72 virgin females were obtained at random from each of the four experimental populations. On day 11 of their life cycle, these females were placed in adult competition vials in groups of 16, along with 16 males taken randomly from the LH_M_ population, for a period of 2 h, during which time all females were observed to have mated once. Males were removed from the vials, and after 2 d in the incubator, females were transferred to individual oviposition vials containing fresh medium (with a scored surface) for a period of 18 h before being discarded. The number of eggs laid in each vial was counted and the mean fecundity of the two replicates of each treatment was compared using a *t*-test (with population as the unit of replication). Since the selected trait in these experimental populations was body size, any consistent change in fecundity between the two treatments must be due to a genetic correlation between the two traits.

### Behavioural Assay of Male Mate Persistence towards Females Differing in Body Size

In order to test whether males have mate preferences, a series of behavioural assays were conducted. Nonvirgin flies from both the LH_M_ and LH_M_-*bw^D^* populations were collected on day 11 of their life cycle, and females were sorted by size to isolate large- and small-bodied individuals. Pairs of female flies differing in eye colour (to aid individual identification) were placed into small, adult competition vials (test tubes) in all possible combinations of body size. After a 1-h anaesthesia-recovery period, a single unanaesthetized adult male fly was added to each test tube, which were then placed on their sides in a well-lit room. Over the course of 11 sessions, spaced 40 min apart, the male in each test tube was observed. Male persistence behaviour was defined as being located within 5 mm of a female and oriented towards her [53]–[55]. Data on the frequency of the male persistence behaviour was collected for each type of female in each treatment. A total of 30 replicate test tubes per treatment were scored.

### Assays of Remating Rates for Females Differing in Body Size under “Choice” and “No-Choice” Environments

In these assays, nonvirgin adult female LH_M_ flies were collected on day 11 of their life cycle and sorted by size (see above) to isolate large and small individuals. Females were then placed into one of two types of adult competition vials (a vial containing fresh medium seeded with 6.4 mg of live yeast). In the first, choice experiment, either eight large or eight small red-eyed LH_M_ females were placed into an adult competition vial along with eight randomly collected LH_M_-*bw^D^* females and 16 LH_M_-*bw^D^* males. In the second, no-choice treatment, either 16 large or 16 small red-eyed LH_M_ adult females were placed into an adult competition vial along with 16 LH_M_-*bw^D^* males. These vials were kept in the incubator (on their sides) for 24 h, at which time males were removed. The vials, containing females only, were then returned to the incubator for an additional 24 h. Remating rates were assayed by placing all females into individual oviposition vials (test tubes) containing fresh, scored medium for the purpose of measuring the paternity of her offspring. Eighteen hours later, the adult flies were discarded, and the test tubes containing eggs were incubated for 11 d. At this time, the presence and number of red-eyed and brown-eyed progeny in each brood were scored to ascertain whether the female had remated. The proportion of females in each adult competition vial that produced brown-eyed offspring (indicating a remating event) was recorded. To examine remating rates in relation to female body size and treatment, we constructed GLMs that used a logit link function and binomial error distribution, where the number of females that remated is the dependent variable and the total number of females assayed is the binomial denominator. We tested whether male mate preferences caused the tails of the distribution of female lifetime fecundity to regress towards the mean by performing a two-way ANOVA, with body size, remating treatment, and their interaction as predictor variables. A significant interaction term (that was associated with a smaller difference between the mean fecundity of large and small females when male preference was possible) would indicate that the tails of the fecundity distribution had regressed toward the mean. Each type of remating assay was repeated twice. The first, choice assay was comprised of ten adult competition vials (the unit of replication) for each body size treatment, whereas the second replicate was comprised of 38 adult competition vials in the large body size treatment and 37 in the small body size treatment. Both replicates of the no-choice assay were comprised of 15 adult competition vials for each body size treatment.
